# ﻿Going deeper and further: a range and depth extension for the deep-sea feather star *Paratelecrinuscubensis* (Carpenter, 1881) (Comatulida, Atelecrinidae), first record from the Western Pacific

**DOI:** 10.3897/zookeys.1184.110577

**Published:** 2023-11-15

**Authors:** Zijie Mei, Zhongli Sha, Shao’e Sun

**Affiliations:** 1 Department of Marine Organism Taxonomy and Phylogeny, Institute of Oceanology, Chinese Academy of Sciences, Qingdao 266071, China; 2 Laoshan Laboratory, Qingdao 266237, China; 3 Shandong Province Key Laboratory of Experimental Marine Biology, Institute of Oceanology, Chinese Academy of Sciences, Qingdao 266071, China; 4 University of Chinese Academy of Sciences, Beijing 100049, China

**Keywords:** Magellan Seamounts, new record, phylogenetic relationships, taxonomy

## Abstract

A specimen belonging to the deep-sea feather-star family Atelecrinidae was collected in April 2018 at the Kocebu Guyot at 1294 m deep. Based on its morphological characters, the specimen was identified as *Paratelecrinuscubensis* (Carpenter, 1881). This species of feather star is restricted to the deep sea and was known only from 12 records from the Bahamas and Cuba at depths of 567–892 m. The data represent the first record from the Western Pacific Ocean and the deepest record known, extending the depth where this feather star has been found to beyond 1000 m. The morphological characteristics of the Kocebu Guyot specimen were essentially identical to the morphology of the neotype, with a slight difference in the dorsal spine at the end of the cirri. The phylogenetic analysis based on the mitochondrial cytochrome c oxidase subunit I (COI), 16S rRNA genes, 28S rRNA genes, and 18S rRNA genes reveal a close relationship of *P.cubensis* with *P.wyvilli*. Results of our molecular phylogenetic analysis are consistent with our morphological identifications. Our find extends the known geographical distribution of the feather star *P.cubensis* to the Western Pacific Ocean and provide insights into deep-sea biodiversity in the Kocebu Guyot.

## ﻿Introduction

In April 2018, the Institute of Oceanology of the Chinese Academy of Sciences (IOCAS) conducted a biodiversity survey in the Kocebu Guyot. Several echinoderms were collected, including crinoids. After examination and identification, one individual was found to be referable to *Paratelecrinuscubensis* (Carpenter, 1881), the type species of *Paratelecrinus*. This species was previously recorded only in deep-sea off the Bahamas and Cuba, and not recorded in the Western Pacific.

The family Atelecrinidae Bather, 1899 is widespread in the Atlantic, Indian, and tropical Pacific Oceans (Messing 2013). The Atelecrinidae have some unique and interesting morphology compared to other feather stars; for example, the basals form an externally visible ring, the cirrus sockets are in 10 or 15 columns, and the ray’s distal part without pinnules is known as the long filament (Messing 2013). According to WoRMS (Messing et al. 2023), this family includes four genera and 12 species.

Bather (1990) established a new family, Atelecrinidae (including *Atelecrinus* Carpenter, 1881). *Jaekelometra* Gislén, 1924 and *Sibogacrinus* A.H. Clark (in A.H. Clark & A.M. Clark), 1967 were removed from this family by Messing (2003) because of their tall basals. Hess and Messing (2011) placed *Atopocrinus* A.H. Clark, 1912 in a new family, Atopocrinidae, but left *Sibogacrinus* in Atelecrinidae, although its centrodorsal cavity is small in comparison.

Messing (2013) revised Atelecrinidae, based on existing and newly collected specimens, and established two new genera, *Adelatelecrinus* Messing, 2013 and *Paratelecrinus* Messing, 2013, and five new species. *Atelecrinuswyvilli* Carpenter, 1882, *A.conifer* A.H. Clark, 1908, and *A.cubensis* Carpenter, 1881 were moved to *Paratelecrinus*, to join with four newly described species in this genus. *Paratelecrinus* has a distinct ligamentous bundle between the centrodorsal and basals. The exterior visible portion of the basals form a shallow chevron- or inverted V-shape. The cirrus sockets have fulcral tubercles similar to *Atelecrinus* and *Adelatelecrinus*, but these tubercles are more developed. Furthermore, in contrast to *Atelecrinus* and *Adelatelecrinus*, the aboral surface of the basals in *Paratelecrinus* bears a more strongly and intricately sculpted interradial depression. The basals of *Paratelecrinus* and *Adelatelecrinus* are connected to centrodorsal by ligament bundles, whereas *Atelecrinus* differs from them in the absence of distinct ligament bundles and is restricted to the Atlantic Ocean.

Our study is the first report of *P.cubensis* from Kocebu Guyot, Western Pacific. In addition, we conducted molecular phylogenetic analyses to assess the systematic position of *P.cubensis*. Our study will provide an important for exploring the geographic distribution of *P.cubensis*.

## ﻿Materials and methods

### ﻿Sampling and preservation

**Figure 1.**
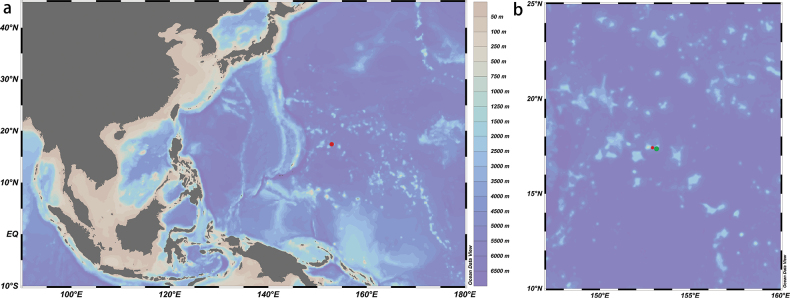
Location of the new record of *Paratelecrinuscubensis***a** map showing the Magellan Seamounts (red dot) **b** map showing the collection location (green dot) and the Kocebu Guyot (red dot).

A single specimen of *Paratelecrinuscubensis* was collected by the submersible remotely operated vehicle (ROV) *FaXian* carried by the R/V *KeXue* during the deep-sea biological survey of Magellan Seamounts by the Institute of Oceanology, Chinese Academy of Sciences (IOCAS). The specimen was collected in April 2018 at a depth of 1294 m at the station FX-Dive177 (17°21′14″N, 153°08′35″E) (Fig. 1a). The specimen is preserved in 70% ethanol, assigned the voucher number M1233, and maintained at the Museum of Marine Biology, Chinese Academy of Sciences (**MBMCAS**).

### ﻿Morphology observation

We examined the specimen using a stereo-dissecting microscope (Zeiss SteREO Discovery V12). We traced photographs to make line drawings, which were completed in Adobe Photoshop 2021 using a graphics tablet. Linear structural features (≥1 mm) of the specimen were measured with digital vernier calipers. For the curvilinear structures which were difficult to measure, a ZEISS Axiocam 506 microscope camera was used to take photographs, and the Leica LAS Image Analysis software was used to conduct the measurements. All measurements were rounded to the nearest 0.1 mm.

See Messing (1997) and Hess and Messing (2011) for general morphology of comatulids and Messing (2013) for morphology of Atelecrinidae. Our methods of measurements, and mode of description, and nomenclature used follow Messing (2013).

### ﻿DNA extraction, sequencing and phylogenetic analyses

All genomic DNA was obtained from pinnules using E.Z.N.A. Tissue DNA Kit according to the manufacturer’s instructions. The TruSeq Nano DNA Sample Prep Kit (Illumina, San Diego, CA, USA) was used to construct the paired-end library with an insert size of 450 bp. The library was sequenced by an Illumina (San Diego, California, USA) HiSeq 4000 platform (2 × 150 bp paired-end reads). The raw sequences were trimmed using Trimmomatic v. 0.39 (Bolger et al. 2014) to obtain about 10 G clean reads. The clean reads were assembled de novo by SPAdes v. 3.10.1 (k-mer = 21–77) (http://bioinf.spbau.ru/spades). The obtained contigs was 504,782 kb. Four mitochondrial gene fragments with GenBank accession numbers OR345518, OR350562, OR660692, and OR660694 (COI, ~1536 bp; 16S, ~1536 bp; 28S, ~738 bp; 18S, ~2064 bp) were extracted from the contigs assembled from the clean reads, which were obtained through Illumina sequencing. Four sequences of *P.wyvilli* were used as reference sequences (COI, KC626573; 16S, KC626665; 28S, KC626853; 18S, KC626759) .

Before phylogenetic analysis, we estimated the intergeneric genetic distance based on the available COI barcoding sequences of two species of *Paratelecrinus* and some genera of the suborder Bourgueticrinina. Kimura-2 parameter (K2P) genetic distances were calculated using MEGA v. 6 (Tamura et al. 2013). For the phylogenetic analysis, the concatenated sequences of COI, 16S, 18S, and 28S fragments of seven species (four families) from Bourgueticrinina and one outgroup from Asterometridae were used (Suppl. material 1, download from NCBI). The nucleotide sequences for COI, 16S, 18S, and 28S genes were all aligned with MEGA v. 6 (Tamura et al. 2013).

Phylogenetic trees were constructed by maximum-likelihood (ML) and Bayesian-inference (BI) analysis. PartitionFinder (Lanfear et al. 2017) was used to select the best partition model. ML analysis was conducted by IQ-TREE web server (Nguyen et al. 2015) under the model automatically with 5000 ultra-fast bootstrap replications (Minh et al. 2013), as well as the Shimodaira-Hasegawa-like approximate likelihood-ratio test (Minh et al. 2013). BI analyses were performed using MrBayes v. 3.2.6 software (Ronquist et al. 2012) under the best-partition model (COI, GTR+G; 16S, GTR+G; 18S, HKY+I; 28S, GTR+G) (2 parallel runs, 5 million generations), in which the initial 25% of sampled data were discarded as burn-in, with a sampling frequency of 100 generations to allow sufficient time for convergence (the standard deviation of split frequencies less than 0.01). The effective sample size (ESS) values for all sampling parameters were checked with Tracer v. 1.7 (Rambaut et al. 2018). The first 12,500 trees were discarded as burn-in, and the remaining trees were used to compute the 50% majority-rule consensus tree and the posterior probabilities (PP).

## ﻿Results

### ﻿Systematics

**Class Crinoidea Miller, 1821**

**Order Comatulida AH Clark, 1908**

**Family Atelecrinidae Bather, 1899**

#### ﻿Genus Paratelecrinus

Taxon classificationAnimaliaComatulidaAtelecrinidae

Messing, 2013

35F16CC7-8CCC-5309-9BDC-234CF04102B2

Paratelecrinus cubensis (Carpenter, 1881).
Antedon cubensis : Pourtalès 1869: 356 (in part); 1878: 214–215 (in part).
Atelecrinus cubensis Carpenter, 1881: 15–19, pl. 1 fig. 7; 1882: 491–492; 1888: 70–72. A.H. Clark 1907: 155. Hartlaub 1912: 281, 386, 484, pl. 14, figs 3, 8, 9.
Atelecrinus pourtalesi : A.H. Clark 1907: 4. H.L. Clark 1941: 13.
Atelecrinus balanoides : A.H. Clark and A.M Clark 1967: 819, 823–831 (in part).
Paratelecrinus cubensis : C.G. Messing 2013: 22–24, figs 9, 10.

##### Material examined.

MBM287771, 1 specimen; Western Pacific, Kocebu Guyot, R/V *KeXue* station FX-Dive177; 17°21′14″N, 153°08′35″E; 1294 m depth, 11 April 2018, hard substrate.

##### Description.

**Figure 2.**
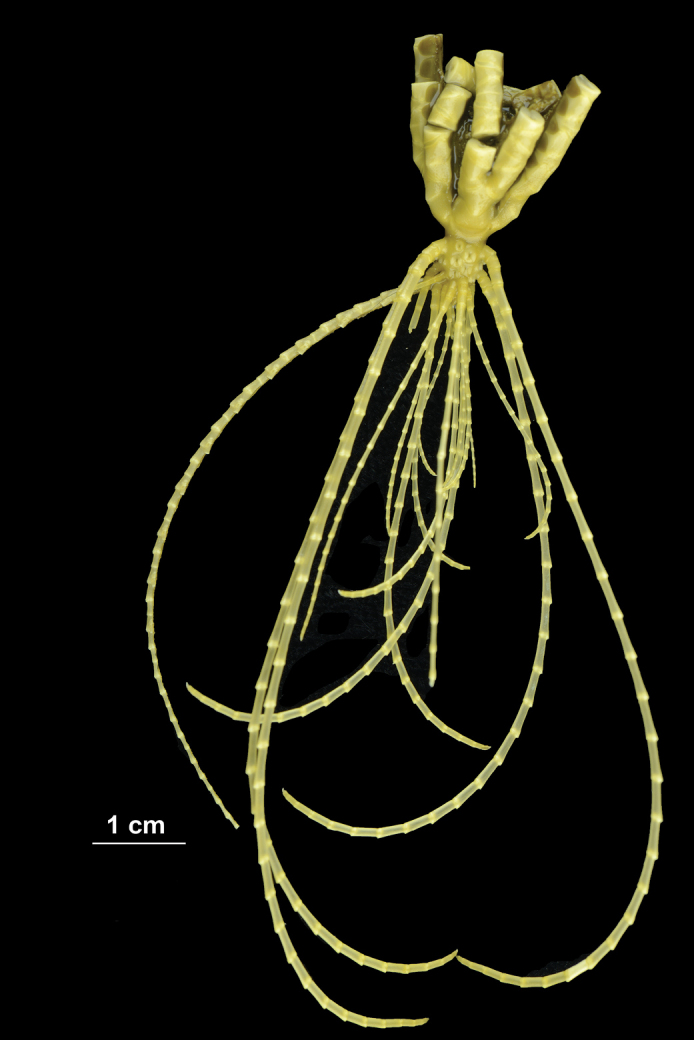
*Paratelecrinuscubensis* from Kocebu Guyot, showing the original color. The first “cirri” from the left is a broken part of a distal arm.

The middle and distal parts of the rays of the specimen are missing, broken off at IIbr3, IIbr6, and IIbr9 (Fig. 2).

**Figure 3.**
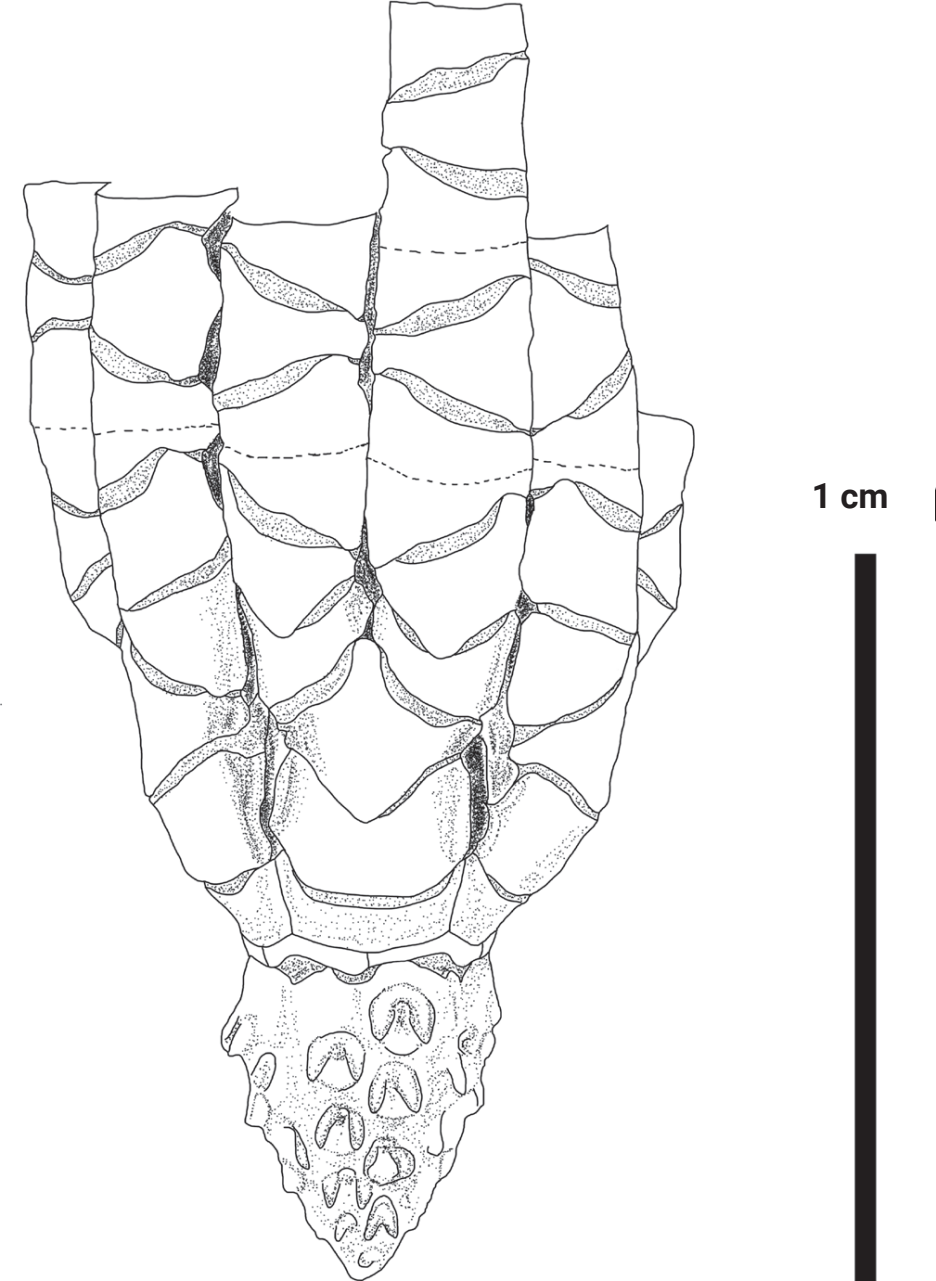
*Paratelecrinuscubensis* from Kocebu Guyot: Centrodorsal and ray base.

**Figure 4.**
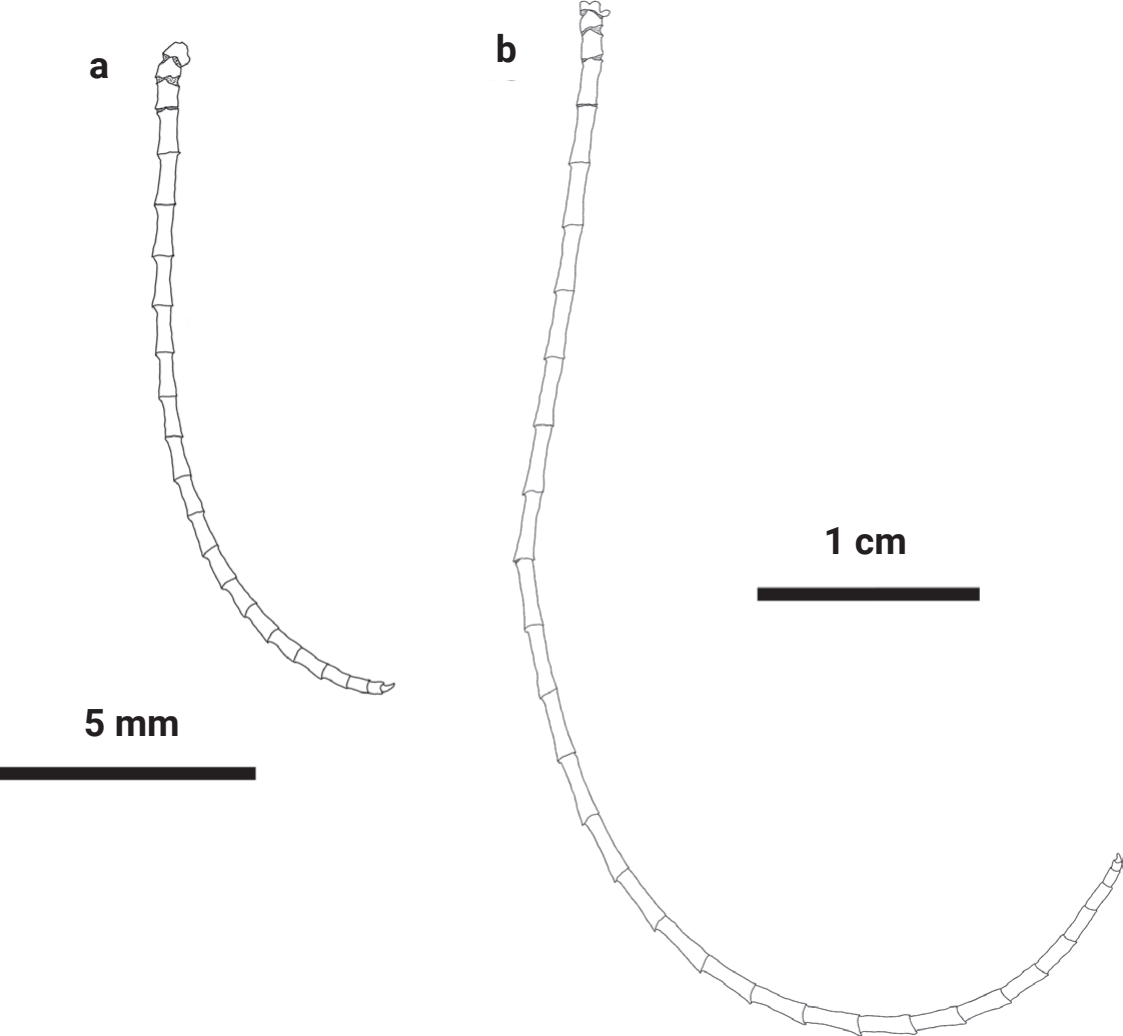
*Paratelecrinuscubensis* from Kocebu Guyot **a** apical cirrus **b** peripheral cirrus.

**Figure 5.**
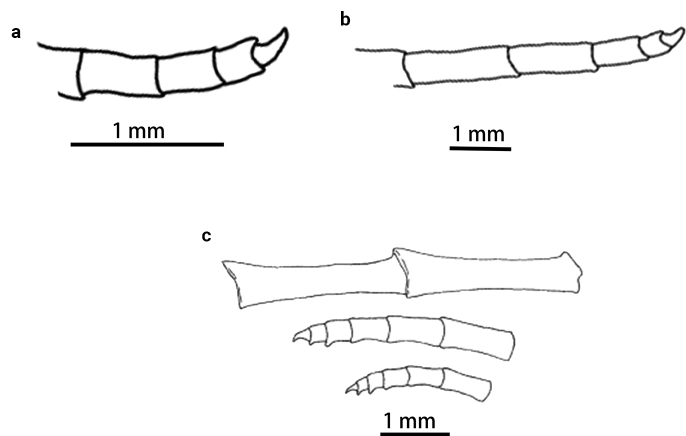
*Paratelecrinuscubensis* from Kocebu Guyot **a** tips of apical cirrus **b** tips of peripheral cirrus **c** two middle cirrals (upper) and tips of two cirri (middle, lower) (modified from Messing 2013).

Centrodorsal conical, base diameter 3.0 mm, H/D 1.3; interradial margin with U-shaped depression (Fig. 3). Cirrus sockets distributed in 10 columns, with strong fulcral tubercles. Cirri XL, only one complete peripheral cirrus (Fig. 4b) 69.1 mm long and with 31 segments; c1–3 short and cirrals gradually increasing in length, with expanded distal ends; c8–c13 longest, L/W 6.7; penultimate cirral squarish, with opposing spine weak or absent, terminal claw curled (Fig. 5b). Apical cirrus of 22 segments, 15.0 mm long; c5–c7 longest, L/W 3.4 (Fig. 4a).

**Figure 6.**
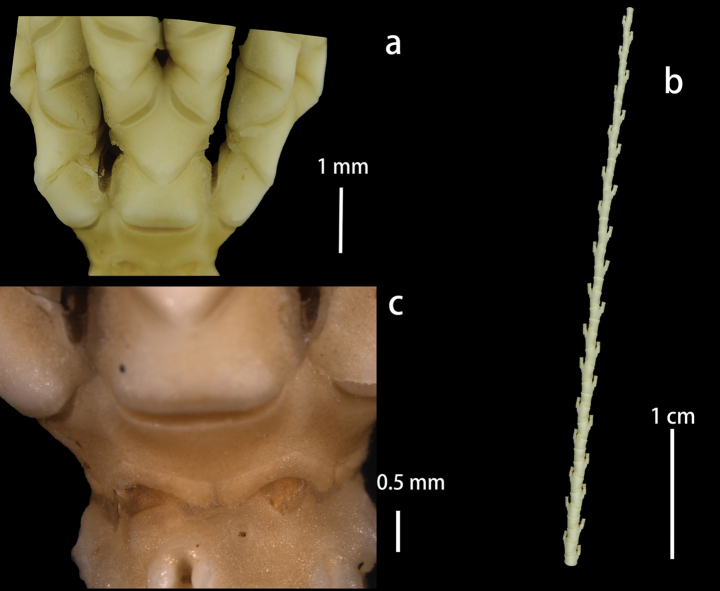
*Paratelecrinuscubensis* from Kocebu Guyot **a** IBr and IIBr flanges **b** distal arm part **c** basals and radials.

Basals form a complete ring, separated from centrodorsal by distinct ligamentous bundles, especially interradially; externally visible portion of basals swollen interradially and then narrowing laterally, with a concave lower edge interradially and an overall inverted V-shape (Fig. 6c). Radials are very short, W/L 3.2. Lateral margin of radials clearly visible, separating adjacent brachitaxes (Fig. 6c).

Arms 10, 2.9–8.3 mm long (Fig. 3). First brachitaxes and proximal rays with moderately developed synarthrial tubercles. Ibr1 rectangular, with V-shaped distal margin and thin projections on lateral margins, W/L 2.5. Iax2 rhombic, laterally margin with wing-like flange, lower edge distinctly convex, W/L 1.1. IIbr1, W/L 2.7, outer lateral margin longer and slightly curled inward (Fig. 6a). IIbr2, W/L 1.3, irregularly square, outer lateral margin longer, distal margin wider, proximal margin V shaped, with sufficient clearance from adjacent IIbr2. IIbr_3+4_ longer interiorly, W/L 1.2, 1.7 mm across. Middle brachials wedge-shaped, W/L 1.8. Distal brachials wedge-shaped and longer than wide, with distal ends slightly raised, W/L 0.6 (Fig. 6b). Syzygies at (3+4), (6+7), (9+10).

##### Distribution.

Previous records of *P.cubensis* have been only collected in the deep sea off the Bahamas and Cuba (567–892 m) (Messing 2013). The current study is the first report of *P.cubensis* from Kocebu Guyot, which extends the known geographical distribution of this species to the Western Pacific Ocean (Fig. 1). Furthermore, the new record is the deepest known observation of *P.cubensis*; at 1294 m, this observation extends the depth of this feather star beyond 1000 m.

McClain and Hardy (2010) have suggested that bathymetric gradients may impose limitations on the range of species compared to horizontal distances, and that the geographic distribution of species may be more frequent where water depths are deeper. Based on this hypothesis, the large gap in the geographic distribution of *P.cubensis* seems reasonable. Furthermore, biodiversity correlates with latitude, showing patterns of tropical peaks and polar declines in species richness (Mannion et al. 2014). The collection site is consistent with the latitudinal distribution of previous records, which supports the very large geographic distribution of *P.cubensis*.

### ﻿Barcoding, phylogenetic relationships and taxonomic implication

The intergeneric genetic distance (K2P) of the suborder Bourgueticrinina is established based on the COI gene (Table 1). The intergeneric distances within Bourgueticrinina range from 6.0% to 18.1%. *Paratelecrinuscubensis* most closely related to *P.wyvilli*, with a *p*-distance of 6.0%; this supports our morphological identification of our Kocebu Guyot specimen.

**Table 1.** The genetic distance of COI gene (K2P) within *Bourgueticrinina* species.

	1	2	3	4	5	6
1 *Paratelecrinuscubensis*						
2 *Paratelecrinuswyvilli*	0.06					
3 *Adelatelecrinusvallatus*	0.07	0.088				
4 *Phrynocrinusnudus*	0.146	0.157	0.159			
5 *Porphyrocrinusverrucosus*	0.148	0.159	0.161	0.120		
6 *Monachocrinus* sp. BATHY91	0.148	0.157	0.144	0.157	0.181	

**Figure 7.**
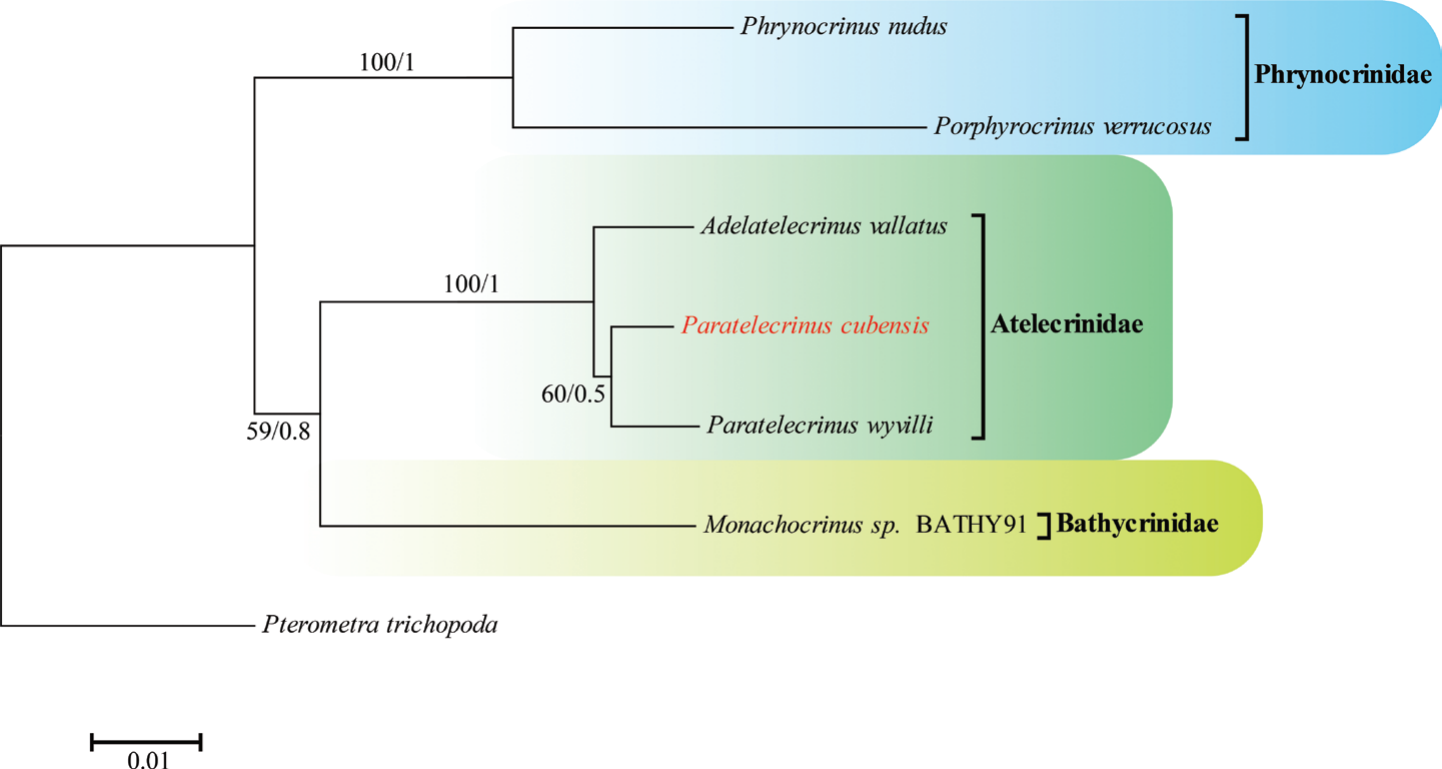
Maximum-likelihood (ML) and Bayesian-inference (BI) trees of Bourgueticrinina using combined COI, 16S, 18S, and 28S sequences. The number at each node represents bootstrap values (BP) (left) and Bayesian posterior probability (BPP) (right). *Paratelecrinuscubensis* is highlighted in red

The phylogenetic tree derived from BI and ML analyses shows essentially the same results (Fig. 7). *Paratelecrinusnudus* and *P.verrucosus* are sister to each other, suggesting that Phrynocrinidae is monophyletic. Atelecrinidae, which groups with *Monachocrinus* sp. BATHY91 and Phrynocrinidae, clusters into a single branch. Within Atelecrinidae, *P.cubensis* and *P.wyvilli* formed the sister group, and then clustered together with *Adelatelecrinusvallatus*.

## ﻿Discussion

In summary, the spoon-shaped aboral fossa in the basals of *Paratelecrinus* species is unique from other genera of Atelecrinidae. The main morphological features of our Western Pacific specimen collected in the Western Pacific are consistent with the neotype (Messing 2013). However, one difference is apparent; the distal three cirrals of the neotype have weak dorsal spines, whereas our specimen has very weak, if not altogether absent, dorsal spines on the distal cirrals (Fig. 5).

*Paratelecrinuscubensis* (10 columns) differs from *P.orthotriremis*, *P.laticonulus*, *P.conifer* and *P.telo* (15 columns) in the number of cirri arrangements. In contrast to other *Paratelecrinus* species, *P.amenouzume* has the weak synarthrial swelling between Ibr1 and Iax2, as well as IBr2 being proportionately more elongate. Consequently, *P.cubensis* is more similar to *P.wyvilli*, but the basal of *P.wyvilli* forms an almost highly coherent narrow band rather than narrowing laterally and expanding at the end; more conspicuous is the absence of wing-like lateral flanges in Iax2 (Messing 2013).

This is the first time for *P.cubensis* recorded in the Western Pacific, as the species was previously known from only the Bahamas and Cuba.

Although there is a lack of molecular information for Atelecrinidae, the relationship between Atelecrinidae, Phrynocrinidae, and Bathycrinidae, as shown in our phylogenetic analysis, is consistent with the study by Hemery et al. (2013). Despite low-level support, *P.cubensis* and *P.wyvilli* clustered together. In conclusion, the taxonomic status of *P.cubensis* from Kocebu Guyot in the Western Pacific, is well established, and the results of our phylogenetic analysis are consistent with our morphological identification.

## Supplementary Material

XML Treatment for Paratelecrinus

## ﻿Additional information

### Author ORCIDs

Zijie Mei https://orcid.org/0009-0002-6818-0565

Zhongli Sha https://orcid.org/0000-0002-2192-3758

Shao’e Sun https://orcid.org/0009-0003-5774-6209

### Data availability

In accordance with FAIR principles, the COI and 16S sequence dataset in this study (Supplementary Materials) can be found in the Supplemental Materials and is available in GenBank.

## Supplementary materials

Supplementary material 1 Sequence information needed to construct phylogenetic trees Data type xlsx https://binary.pensoft.net/file/937353This dataset is made available under the Open Database License (http://opendatacommons.org/licenses/odbl/1.0/). The Open Database License (ODbL) is a license agreement intended to allow users to freely share, modify, and use this Dataset while maintaining this same freedom for others, provided that the original source and author(s) are credited.Zijie Mei, Zhongli Sha, Shao’e Sun
